# Tablet-based adaptation and administration of the Castles and Coltheart Reading Test 2 for a large longitudinal study

**DOI:** 10.1371/journal.pone.0239420

**Published:** 2020-09-18

**Authors:** Clair Bennett, Meabh Cullinane, Shannon K. Bennetts, Jasmine Love, Naomi J. Hackworth, Fiona K. Mensah, Sheena Reilly, Jan M. Nicholson, Elizabeth M. Westrupp

**Affiliations:** 1 Judith Lumley Centre, La Trobe University, Melbourne, Australia; 2 Murdoch Children’s Research Institute, Melbourne, Australia; 3 Parenting Research Centre, Melbourne, Australia; 4 Department of Paediatrics, The University of Melbourne, Melbourne, Australia; 5 The Royal Children’s Hospital, Melbourne, Australia; 6 Griffith University, Gold Coast, Queensland, Australia; 7 Deakin University, Melbourne, Australia; Universidad Autonoma de Madrid, SPAIN

## Abstract

Tablet-adapted measures provide an efficient, accurate method of data collection for large-scale studies. The Castles and Coltheart Reading Test 2 (CC2) is a standardized paper-and-pencil measure of children’s reading ability. In the current study, the CC2 was administered to 603 children aged 7–8 years via iPad using electronic data capture software. Results indicate the tablet-adapted measure could be reliably administered by non-clinical staff and showed quantitative equivalence, i.e., comparable score distributions, to CC2 normative data. Internal consistency was good for regular and non-word lists. Findings suggest that the tablet-adapted CC2 is a viable tool for large research studies.

## Introduction

Computerized data collection methods are increasingly utilized in clinical and population-based research [1, 2], as they provide a potential means of improving accuracy and efficiency of data collection. Computer-adapted measures overcome several limitations associated with paper-and-pencil alternatives. Importantly, computer adaptation may address possible issues of assessment fidelity, allowing administration by a non-specialist workforce and improving standardization of test administration. Direct data entry and data validation can also be implemented, reducing the amount of time and resources invested in data entry, checking and cleaning [3]. The potential advantages of computer-adapted measures are greater for large-scale studies where data entry and checking processes can become highly resource-intensive. With the development of portable technology, computerized assessments can likewise be conducted on tablet devices—a cheaper and more portable alternative to computers [4].

The portability and reduced costs associated with tablet-based data collection methods are particularly beneficial for studies where assessments are conducted remotely and over dispersed geographical regions [5]. Tablet-based data collection also enhances data security through password-protection and immediate data upload, thus minimizing instances of misplaced or damaged raw data from hard-copy data entry forms. This is particularly important for field studies that require researchers to travel frequently and conduct decentralized data collection. Accordingly, as portable tablet devices become increasingly commonplace, the up-front research costs of tablet-based test administration (i.e., cost of tablets, optimization for tablet format) may be offset by reduced printing and study personnel costs associated with secondary data entry and checking procedures. Whilst some assessments require complex adaptation by tablet or Web application developers with considerable programming expertise, other paper-and-pencil based measures may be more readily adapted with the use of existing electronic data capture technology [6].

Tests of reading, in particular, are frequently text-based and therefore may be more easily adapted to a computerized format compared to other cognitive and developmental assessments that may require more advanced design features, such as complex graphics and reaction time (RT) recording (e.g., tests of visuo-spatial reasoning or psychomotor skills). Yet, the issue of mode equivalence between computer-adapted tests and their paper-and-pencil counterparts should still be considered for any newly adapted measure. One index that may be used to evaluate similarity across testing modalities is quantitative equivalence. Quantitative equivalence is defined as the extent to which means and distributions are similar between test administration modes, and whether the norms associated with one mode of a test may be applied to another mode [7, 8]. This may also be considered a test of criterion validity, i.e., the extent to which outcomes from one method of administration are comparable to those from another method [9].

Research examining mode equivalence of paper-and-pencil and computerized assessments of reading in early childhood is limited and has produced mixed results. For instance, a meta-analysis in kindergarten through 12^th^ grade populations indicated that computer-based versions of reading assessments were generally comparable to their paper-and-pencil counterparts [10]. However, this synthesis of the literature included a broad age range and the vast majority of samples included were of 4^th^ grade students and older. Evidence is mixed with younger children. In a sample of four- to five-year-olds, Carson, Gillon and Boustead [11] similarly found that scores on a computerized version of a phonological awareness assessment were comparable to the paper-based version. By contrast, in a sample of two- to six-year-olds, Neumann and Neumann [12] found that despite a high level of agreement between test modalities assessing several aspects of early literacy (ICCs = .81 –.94), some tablet-based mean scores were higher than their paper-based counterparts. Finally, in a group of first to sixth-graders, Lenhard, Schroeders and Lenhard [13] found main effects of administration modality at the word-reading level, but not at the sentence level or text level when comparing paper-and-pencil and computerized versions of the ELFE II reading comprehension task. The authors also found a very small interaction effect with child grade across all three reading levels. These findings suggest that factors leading to non-equivalence may be measure- and to some extent age-dependent, highlighting the need for validation of any computer- or tablet-adapted measure.

In the present study we assessed children’s word reading using a tablet adaptation of a well-validated paper-based assessment of reading—the Castles and Coltheart Reading Test 2 [CC2; 14]—within a large-scale longitudinal study. The CC2 is a measure of children’s visual word recognition and sounding-out abilities for children between the ages of 6 and 12 years, and includes 40 regular words (e.g., need, plant), 40 irregular words (e.g., island, friend), and 40 non-words (e.g., pleech, framp). Items are presented to the child by an assessor in a fixed order from easiest to hardest, with items from the regular, irregular, and non-word lists presented in a mixed fashion. The assessor records whether the child’s pronunciation of the word is “correct” or “incorrect”, without providing feedback to the child. Stopping rules are applied for each list separately, such that 5 consecutive errors on any list results in discontinuation of that particular list. Items on the other lists continue to be presented until further discontinue criteria are met or until all trials are completed. The CC2 is often used for clinical purposes, administered by clinicians and teachers.

For research purposes, the CC2 can be administered and scored in paper-and-pencil format. The paper-and-pencil form is administered using a set of cards featuring the 120 test items printed in large 36pt font which are presented to the child sequentially. The reverse side of each card is color-coded to assist assessors when applying the stopping rules. The CC2 can also be administered using an online program, and both versions of the test are freely available from the developers’ website: the Macquarie Online Test Interface (MOTIF: www.motif.org.au). Although the MOTIF online version of the task is well-suited to clinicians and teachers working with individual children, there are limitations to its use in a research setting. Firstly, administration of the online task requires internet connectivity, which may be impractical for certain types of field work research. Secondly, the output of the results can only be exported in portable document format (PDF) and not in a more convenient tabular format such as a comma-separated file (CSV). Researchers are therefore still required to manually enter data following test administration.

In the current study, we sought to address these limitations by developing a computerized version of the CC2 that was suitable for large-scale data collection and could be administered offline using a tablet device. Advantages of the tablet-based version included circumventing the need for manual data entry, and the ability to use branching logic (i.e., automated skipping that customizes the sequence of questions based on participants’ previous responses) to apply discontinue rules. This administration approach reduced the burden on assessors to track errors across separate word lists, thereby ensuring compliance of examiners with testing instructions. Nonetheless, it is important to ensure that any changes in presentation modality of existing measures do not compromise the reliability of the measure or introduce systematic biases to test administration. This paper therefore aims to (i) describe the method by which the CC2 was adapted for administration via tablet, (ii) examine criterion validity (i.e., equivalence of administration method) and reliability of the tablet administered data compared to normative data from the paper-and-pencil based version of the CC2, and (iii) detail the process for assessing and maintaining inter-rater reliability of the CC2 on a large-scale study with non-clinician field staff.

## Method

### Study setting and participant selection

Participants were 603 children who completed home-based assessments as part of a longitudinal study conducted in Victoria, Australia [15]. EHLS at School is the school-aged follow-up of a cluster randomized controlled trial (the Early Home Learning Study, EHLS) [16] which evaluated an early childhood parenting intervention when the child was aged between 12 and 36 months. Parents were offered participation in the trial if the family had one or more risk factors for poor child development (e.g., young parent, single parent, low parental education). In the EHLS at School follow-up study, we sought to assess children at around the age of 7.5 years. Written informed consent was obtained from parents on behalf of each study child. The study protocol was approved by the La Trobe University Human Ethics Committee (No. 15–028), the Victorian Department of Education and Training (DET, formerly the Department of Education and Early Child Development) Research Committee, and the Catholic Education Offices of Ballarat, Melbourne, Sale and Sandhurst.

### Measure adaptation

**Fig 1:**
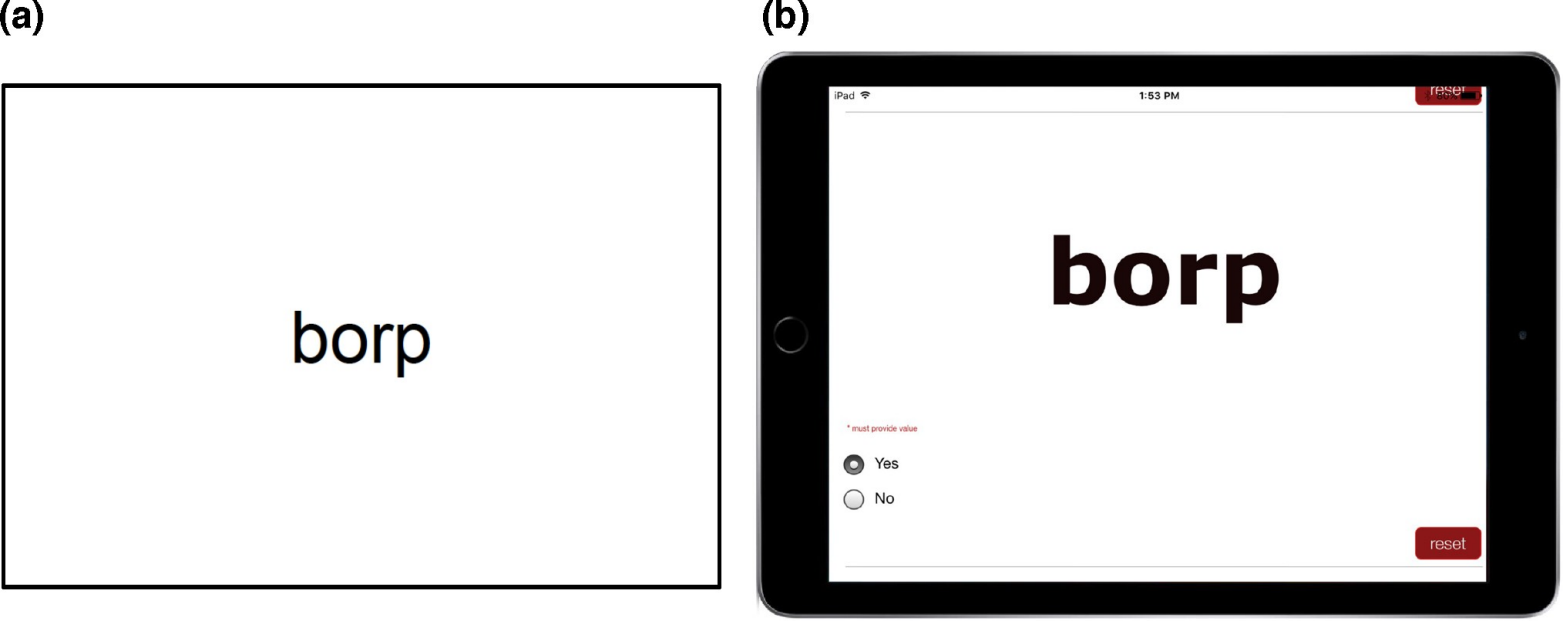
(a) CC2 card presentation, and (b) tablet-based CC2. Each stimulus occupies the entire screen, and the assessor scrolls down to access the subsequent stimulus.

With the permission of the authors [14], the CC2 was adapted for use with REDCap [17] hosted at La Trobe University, and administered on an iPad. REDCap is a secure web application for surveys and databases that supports offline and online data entry. To ensure that presentation closely resembled the paper-and-pencil version of the measure, word lists were coded in an 80-pt font (HTML code) so that each word was singularly presented on the tablet screen with participants unable to see prior or subsequent items (see Fig 1).

### Training and administration procedures

The CC2 was administered by trained research assistants (hereafter, “assessors”). Three assessors were initially trained to score children’s spoken word responses according to audio recordings of word pronunciation provided on the MOTIf website. Inter-rater reliability was estimated using mean Kappa coefficients for each assessment (examining item-by-item agreement). Sufficient inter-rater reliability (i.e., Kappas ≥ .80) was initially established between three assessors. Disagreements regarding pronunciation were resolved via group discussion and through consultation with a senior researcher from the MOTIf group. Five additional assessors were then trained to undertake CC2 administration, with data from five of the previously coded assessments used as the benchmark for establishing inter-rater reliability of the new assessors. If assessors did not attain acceptable reliability (Kappa ≥ .80), feedback and further training were provided. All assessors completed “refresher training” every 3 months to ensure consistency and accuracy between scorers and to minimize rater drift across the 31 months of the study. Refresher training required assessors to review audio pronunciations on the MOTIf website and to score additional training videos to Kappa ≥ .80. This was also an opportunity to identify and clarify the pronunciation of challenging words and receive direct feedback on completed assessments.

With the exception of testing modality, all aspects of test administration were conducted according to the procedures for the paper-and-pencil version. Assessors provided each participant with standardized verbal instructions for the task. Then for each word trial, assessors presented the iPad screen and the child was instructed to read each word aloud. Items were presented sequentially, and the assessor scored the item “yes” for a correct response or “no” for an incorrect response on the iPad using the radio buttons visible in Fig 1B. Scoring was conducted by turning the iPad away from the child and scoring the item out of the child’s view, before scrolling to the next item. Individual items were designed to fit the width of the iPad screen to ensure that an assessor could scroll to the next item—concealing the score for the previous item—before re-presenting the iPad to the child.

As the test was administered by research assistants rather than trained clinicians, assessments were also audio recorded on the iPad to monitor inter-rater reliability and assessor drift. Assessments were conducted over a 31-month period from March 2016 to September 2018. Every 5^th^ CC2 assessment (i.e., 20% of all assessments) was double-scored by a second trained team member. Data were analyzed in R version 3.3.1 [18] and Stata SE Version 14.0 [19].

### Data analysis

For comparisons against normative data on the CC2, children were categorized according to 6-month age bands (from 7;0 to 8;6 years). Six percent of the sample were older than 8 years at assessment and were skewed towards the younger end of the age bracket (M = 8.15 years, SD = 0.14; range = 8.01–8.56 years). The truncated range within this age band precluded meaningful analyses for this group and these children (n = 34) were excluded from the analyses by age band, but were retained for all other analyses. Mean z-scores were determined separately for each age band using the age-specific norms provided by Castles and colleagues [14] to assess whether participants were on average performing at an expected level according to age. Welch’s *t-*tests were conducted with summary data to compare mean scores between published normative data for the CC2 paper-based version from Castles and colleagues [14] and the CC2 tablet-based version across the age bands in order to establish quantitative equivalence between measures. Internal consistency was estimated for the three word lists (regular, irregular, and non-words). Given that each word list progresses with increasing difficulty and has a discontinue rule, many participants are not administered the maximum items. Internal consistency was therefore examined in two ways. First, odd vs. even split-half reliability checks were carried out for each list. The Spearman Brown prophecy formula was applied to the correlations to compute final reliability coefficients. Spearman’s rho is reported, since the data were skewed. Second, Cronbach’s alphas were computed to enable comparison with internal consistency estimates reported in an earlier study [20]. To avoid violation of the assumption of independence [21], these could only be calculated for participants who completed all test items and are therefore likely to over-represent older participants and better readers. Inter-rater reliability was examined using the Kappa coefficient. Inter-rater reliability analyses were conducted on every 5^th^ assessment (approximately 20% of the total sample) and tracked across the period of data collection.

## Results

### Sample characteristics

At the school-age follow-up, mean child age was 7.47 years (SD = 0.27) with a similar proportion of female and male participants (50.1% female, 49.8% male, 0.2% other). For 7% of children (n = 40), the responding parent had not completed high school, 13% (n = 80) were single parent families, 18% (n = 104) were low income families (less than AUD$36,400 per annum), and 31% (n = 189) were families where a language other than English was spoken at home. Average neighborhood socioeconomic status (mean = 988.3, SD = 58.8) was similar to the Australian population (mean = 1000, SD = 50) [22]. Data from the nation-wide Year 3 reading achievement test (NAPLAN) [23] was available for 85% of the sample (n = 511; mean = 464; SD = 99). This corresponds to the 58^th^ percentile for children in the state of Victoria, and indicates the sample were performing at or slightly above the average for same-age children.

### Standardized scores

Mean z-scores for our participants aged 7;0–7;5 (n = 394) were -0.51 for regular words, -0.66 for irregular words, and -0.52 for non-words. Mean z-scores for participants aged 7;6–7;11 (n = 175) were -0.24 for regular words, -0.38 for irregular words, and -0.25 for non-words. As a z-score between -1 and 1 is indicative of a child performing at an average level, these results indicate that the mean written language scores for participants in the current study were within the typically expected range when compared to normative data.

### Means comparisons with normative data

Summary statistics (means, standard deviations) are presented in Table 1 by age band (7;0–7;5 and 7;6–7;11) for regular, irregular, and non-words for the current sample (tablet-based CC2; n = 569) and a previously published normative sample (paper-based CC2; n = 177) from Castles and colleagues [14]. For both age bands, there were no significant differences in mean scores for our sample receiving the tablet-based administration compared to the normative sample who received the paper-based administration.

**Table 1:** T-test results comparing normative with tablet-administered data.

Word Lists		Paper-based CC2		Tablet-based CC2		*t (df)*	*p*
		(*n*)	Mean (SD)	(*n*)	Mean (SD)		
Age band 7;0–7;5							
	Regular words	81	25.2 (9.5)	394	24.6 (11.2)	0.51 (129.80)	.61
	Irregular words	81	13.4 (6.2)	394	13.6 (6.5)	0.22 (119.38)	.82
	Non-words	81	16.5 (10.4)	394	17.5 (12.1)	0.75 (128.44)	.45
Age band 7;6–7;11							
	Regular words	96	27.8 (9.8)	175	27.4 (10.6)	0.32 (209.06)	.75
	Irregular words	96	15.3 (6.1)	175	15.3 (6.5)	0.00 (206.73)	1
	Non-words	96	20.0 (11.4)	175	19.7 (12.4)	0.17 (209.75)	.86

Age band = years; months; Paper-based CC2 = Castles and Coltheart 2 means and SDs of published normative sample [14]; Means refer to accuracy scores on each word list; 34 children from the tablet-based sample were not included as they were over the age of 8 years at assessment.

### Internal consistency

For the current sample, internal consistency was acceptable for the regular words and non-words lists. For regular words, Spearman’s rho (split-half reliability) was .90 for the full sample (n = 603) and Cronbach’s alpha was .79 for the reduced sample who completed the list (n = 378). For non-words, Spearman’s rho was .89 (full sample) and Cronbach’s alpha was .83 (n = 224 who completed). Internal consistency was poor for the irregular word list with a Spearman’s rho of .62 (full sample) and Cronbach’s alpha of .59 (n = 33 who completed).

### Inter-rater reliability

Across assessors and over time Kappa coefficients ranged from .74 to 1.0 with a mean of .91. According to guidelines outlined by Landis and Koch [24], this represents “substantial” to “almost perfect” inter-rater reliability across the duration of the study. A simple linear regression was calculated to assess changes in reliability over the course of the study using days from the first assessment as the independent variable and reliability as the dependent variable. The regression equation was not significant (*F* (1, 120) = 1.57, *p* = .21), with an R^2^ of .01, indicating no evidence of significant change in inter-rater reliability over time.

## Discussion

The aims of this paper were to describe the tablet-based adaptation and administration of a standardized developmental measure of reading, report preliminary psychometric findings, and describe the methodological approach to maintaining inter-rater reliability across a large-scale study. Tablet-based administration was adopted to economize on time and monetary costs associated with paper-and-pencil based tasks, and with the additional aim of mitigating the risk for data entry errors. We developed the assessment within existing data capture technology (REDCap), enabling us to automate the required discontinue rules.

To assess quantitative equivalence between the tablet-adapted CC2 and the paper-based measure, we examined z-scores and conducted means comparisons with published norms for children in our sample aged 7;0 to 7;11. Mean z-scores were within the expected range for age band, while raw score means and standard deviations were similar to the normative sample for children. Together, these findings suggest quantitative equivalence between the tablet-based version of the test and the paper-and-pencil version of the CC2, with no evidence of age effects between test modalities for the two age bands we examined. Nonetheless, it is important to note that, although small, age effects on equivalence have been observed for some tests of reading [e.g., 13] and as such these results do not conclusively demonstrate equivalence for age bands not tested.

Internal consistency for our tablet-based version of the CC2 was good to excellent for the regular words and non-words subscales, but poor for irregular words. Only one other study has reported internal consistency for the CC2, with a sample of 8 to 12-year-old children (n = 30) with reading difficulties. Moore and colleagues [20] reported Cronbach’s alphas for the regular (.85) and non-words lists (.85) that were similar to those achieved in our tablet-based administration. However, their Cronbach’s alpha of .94 for the irregular words list was considerably higher than the alpha of .59 we found based on the subsample of 33 children who completed the irregular words list.

While it is possible that the tablet-based administration adversely affected internal consistency for the irregular words list, several other factors may account for this finding. Irregular words are words that lack consistent grapheme-phoneme correspondence rules and can vary greatly in terms of their spelling-to-pronunciation transparency [25]. In contrast to the regular words and non-words which require the child to apply knowledge of spelling-to-sound correspondence rules (a non-lexical process), the correct reading of an irregular word requires the child to be familiar with that word (i.e., stored in a mental lexicon) [26]. In our relatively young sample compared to Moore and colleagues [20], greater internal inconsistency in the irregular word list may reflect a greater degree of guessing by children with less well-developed lexicons.

Differences in sample diversity may also contribute to the differences between the two studies on this word list. For example, children in Moore and colleagues’ study all had poor reading ability, while our participants scored at or slightly higher than the state average for age on the nation-wide reading assessment (NAPLAN), with a distribution around this mean that was typical for age. Around a third of our sample were from households with non-English speaking backgrounds, which would further increase variability in the sample. Young children from multi-language backgrounds may have relatively less developed lexicons as a result of less frequent exposure to English language words at home, compared with their monolingual peers [27, 28]. It is therefore possible that the combination of greater diversity and younger age in our study contributed to poorer internal consistency compared to Moore and colleagues, due to a wider distribution of lower scores, whereas the more representative nature of our sample with respect to reading ability may have resulted in a wider distribution of higher scores. Further research involving larger samples and greater age diversity is required to determine whether the tablet-based administration of the irregular words component is internally valid in its current form.

Finally, inter-rater reliability was examined for the tablet-based format of the CC2, and found to be excellent across the duration of the study, with no evidence of significant rater drift over 31 months of assessments. Inter-rater reliability has not previously been reported for this measure. Our findings suggest that the CC2 can be reliably administered by non-clinicians for research purposes within the defined context in which training was completed prior to data collection, reliability was assessed repeatedly and “refresher” training with feedback was conducted on a regular basis. While this model may be less feasible in clinical practice settings, the benefits are substantial for large-scale research endeavors and justify the resource costs of using this rigorous process.

Our findings should be considered within the context of several limitations. This research was conducted within the context of a larger study that was not primarily established to validate the tablet-based CC2, and as such did not employ the gold standard design for assessing equivalence of test modality (i.e., allocation of children to complete both test modalities in a randomized order). While our findings support the criterion validity of all CC2 subscales and the internal consistency of the regular words and non-words subscales, the narrow age range of our participants precludes generalizability to the full population for whom the CC2 has been designed (i.e., ages 6–12 years). Additionally, while our findings demonstrate support for tablet-based administration of this measure of word reading, this may not apply to other types of reading assessment (e.g., text reading, comprehension test).

Computerized assessment is not without limitations. For instance, potential data loss may occur due to equipment failure, or in some cases, testers may incorrectly press a button and lose the ability to immediately correct the error. Familiarity with tablets and computers and the degree of interaction required should also be considered when assessing young children. Accordingly, while the potential advantages of computer-adapted measures are many for large-scale studies including costs and time savings, they may equally not be appropriate for all studies.

Finally, a notable advantage of electronic test administration is the potential to collect time-related data such as reaction time and total completion time. The CC2 has not previously assessed these features of children’s responding which may shed light on difficulties in processing and articulating written words. The tablet version employed for this study offers a platform for further development and exploration of the clinical utility of time-related data.

In the current study, adaptation of the CC2 was accomplished using existing data capture technology (REDCap) and demonstrated good reliability and similar score distributions compared with normative data, suggesting that successful adaptation of a measure which is psychometrically similar to the paper-and-pencil version can be achieved. We also demonstrated that quality assurance processes can ensure excellent inter-rater reliability despite the use of multiple raters over a lengthy period when rater drift could normally be expected. Frequent training and continued monitoring were essential to this. Our findings support the feasibility and utility of tablet-based assessment as a potential means of improving efficiency and accuracy of data collection for large-scale developmental studies.
